# **α**-Catenin expression has prognostic value in local and locally advanced prostate cancer

**DOI:** 10.1038/sj.bjc.6690381

**Published:** 1999-05-01

**Authors:** S Aaltomaa, P Lipponen, M Ala-Opas, M Eskelinen, V-M Kosma

**Affiliations:** Departments of Urology, andSurgery, andPathology, Kuopio University Hospital; Departments of Pathology and ^4^Forensic Medicine, University of Kuopio, PO Box 1627, FIN-70211, Kuopio, Finland

**Keywords:** α-catenin, prostate neoplasm, prognosis

## Abstract

Normally functioning cell–cell adhesion plays an important role in the maintenance of tissue architecture and cell cohesion. E-cadherin is an important adhesion molecule of epithelial cells. In many types of cancer the expression of E-cadherin is reduced leading to increased risk of disease progression. α-Catenin is one of the intracellular elements of the E-cadherin–catenin complex. The abnormalities in the expression of α-catenin seem to associate with malignant cellular features and disease progression in prostate cancer. To further analyse the significance of α-catenin expression, we studied 215 cases of prostate cancer by immunohistochemistry and the results were related to other known prognostic factors and patient survival during a mean follow-up period of 13 years. α-Catenin expression was down-regulated in 19% of the cases and 3% of the tumours were totally α-catenin-negative. The abnormal α-catenin expression and cytoplasmic signal were significantly linked with high T-category, metastatic disease, high Gleason score, perineural growth, high mitotic rate, high S phase fraction and DNA aneuploidy (*P* < 0.05 for all). In the survival analysis, reduced α-catenin expression (*P* = 0.06) and cytoplasmic signal (*P* = 0.04) were related to unfavourable patient outcome. In the multivariate analysis, including TM-classification and Gleason score, α-catenin expression had independent prognostic value in T1–2 M0 tumors. In the M0 tumours, abnormal α-catenin signal was independently associated with recurrence-free survival as well. The results indicate that down-regulation of α-catenin is related to several malignant cellular features, and it seems to have prognostic significance in the early phases of cancer progression. We suggest that α-catenin expression can provide prognostic information in early prostate cancer. © 1999 Cancer Research Campaign


*British Journal of Cancer* (1999) **80**(3/4), 477–482
© 1999 Cancer Research Campaign
Article no. bjoc.1998.0381

### α-Catenin expression has prognostic value in local and locally advanced prostate cancer

**S Aaltomaa[1], P Lipponen[2], M Ala-Opas[1], M Eskelinen[3] and V-M Kosma[2,4]**

Departments of [1]Urology, [3]Surgery and [4]Pathology, Kuopio University Hospital, Departments of [2]Pathology and [4]Forensic Medicine, University of Kuopio, PO Box 1627, FIN-70211, Kuopio, Finland

**Summary** Normally functioning cell–cell adhesion plays an important role in the maintenance of tissue architecture and cell cohesion. E-cadherin is an important adhesion molecule of epithelial cells. In many types of cancer the expression of E-cadherin is reduced leading to increased risk of disease progression. α-Catenin is one of the intracellular elements of the E-cadherin–catenin complex. The abnormalities in the expression of α-catenin seem to associate with malignant cellular features and disease progression in prostate cancer. To further analyse the significance of α-catenin expression, we studied 215 cases of prostate cancer by immunohistochemistry and the results were related to other known prognostic factors and patient survival during a mean follow-up period of 13 years. α-Catenin expression was down-regulated in 19% of the cases and 3% of the tumours were totally α-catenin-negative. The abnormal α-catenin expression and cytoplasmic signal were significantly linked with high T-category, metastatic disease, high Gleason score, perineural growth, high mitotic rate, high S phase fraction and DNA aneuploidy ($P < 0.05$ for all). In the survival analysis, reduced α-catenin expression ($P = 0.06$) and cytoplasmic signal ($P = 0.04$) were related to unfavourable patient outcome. In the multivariate analysis, including TM-classification and Gleason score, α-catenin expression had independent prognostic value in T1–2 M0 tumors. In the M0 tumours, abnormal α-catenin signal was independently associated with recurrence-free survival as well. The results indicate that down-regulation of α-catenin is related to several malignant cellular features, and it seems to have prognostic significance in the early phases of cancer progression. We suggest that α-catenin expression can provide prognostic information in early prostate cancer.

**Keywords**: α-catenin; prostate neoplasm; prognosis

E-cadherin is a transmembrane protein in epithelial cell–cell interactions at adherence junctions and is linked with the cytoskeletal matrix through interactions with α-catenin (Gumbiner and McCrea, 1993; Breen et al, 1995). The expression of the homotypic cell adhesion molecule E-cadherin is reduced in many types of cancer (Morton et al, 1993, 1995; Rimm et al, 1995; van der Wurff et al, 1997). The loss of this protein may be associated with metastasis because alteration of its function is required for invasion in vitro (Behrens et al, 1989; Frixen et al, 1991; Vleminckx et al, 1991; Breen et al, 1995). The decreased expression has also been related to more aggressive tumour behaviour in vivo (Rimm et al, 1995; Paul et al, 1997; Richmond et al, 1997; van der Wurff et al, 1997). It is likely that the loss of downstream effector elements in the cadherin adhesion cascade (Gumbiner et al, 1993; Breen et al, 1995) may also disrupt cell–cell interactions and thereby promote invasion, but direct evidence is still scanty. One such effector element is α-catenin (Gumbiner et al, 1993; Breen et al, 1995), a cytoplasm protein related to vinculin that is associated in vivo with E-cadherin. Catenins play an important role in the dysfunction of the cell adhesion complex and mechanisms of inactivation of the cadherin–catenin pathway include LOH (loss of heterozygosity), gene deletions and gene promoter hypermethylation (Morton et al, 1993; Vermeulen et al, 1996; Crundwell et al, 1997).

Previous studies indicate that E-cadherin expression correlates inversely with tumour grade and stage in various cancers (Breen et al, 1995; Rimm et al, 1995; van der Wurff et al, 1997), including prostate cancer (Umbas et al, 1994; Crundwell et al, 1997; Murant et al, 1997; Paul et al, 1997; Richmond et al, 1997), and it seems to have prognostic value (Umbas et al, 1994; Richmond et al, 1997). The significance of α-catenin is not completely understood in prostate cancer progression (Crundwell et al, 1997; Murant et al, 1997; Paul et al, 1997; Richmond et al, 1997; Umbas et al, 1997). Initial studies (Crundwell et al, 1997; Richmond et al, 1997) based on small number of prostate cancer cases, reveal that up to 40% of the tumours may show abnormal α-catenin expression. In addition, this down-regulation correlates to disease progression (Crundwell et al, 1997; Richmond et al, 1997; Umbas et al, 1997). Prompted by the above mentioned observations (Crundwell et al, 1997; Shimazui et al, 1997; Umbas et al, 1997; Zsciesche et al, 1997) we (a) analysed the expression of α-catenin in unselected large series of prostate cancer and (b) related the results to other known prognostic factors and patient survival.

*Received 27 May 1998*
*Revised 5 October 1998*
*Accepted 22 October 1998*

*Correspondence to:* P Lipponen

### MATERIALS AND METHODS

### Patients

The current study comprised 215 patients with prostate cancer diagnosed and treated at the Department of Urology, Kuopio

University Hospital, between 1973 and 1992. The mean observation period was 13 years [standard deviation (s.d.) 3, range 8–21] and the mean age of patients at presentation was 72 years (s.d. 8, range 40–89). The cohort was not entirely consecutive since sufficient tumour specimens for immunohistochemistry were not available in all cases. Tumour-metastasis classification was done according to UICC standards (UICC, 1978). Radical prostatectomy was rarely done during the study period, which explains why the node (N) classification was not available. The patients were treated by orchiectomy in 100 cases, other endocrine therapy was used in 70 cases, radical prostatectomy or radiation therapy was done in nine cases and careful follow-up only was used in 76 cases. The follow-up reviews were done at 3-month intervals during the first 2 years, and thereafter at 6-month intervals. At the time of diagnosis, 61/215 (28%) cases had distant metastasis. The causes of death were verified from the patient files, autopsy reports and from the files of Finnish Cancer Registry.

### Histological methods and flow cytometry

The histological samples were core needle biopsies or TURP specimens fixed in buffered formalin (pH 7.0), embedded in paraffin, sectioned at 5 μm and stained with haematoxylin and eosin. The histological differentiation of tumours was evaluated as described by Gleason (1977). The perineural growth was categorized into two groups; present or absent. The mitotic figures were identified and calculated from the most actively proliferating area in the section (Vesalainen et al, 1995). The volume corrected mitotic index method (M/V index) was used, which expresses the number of mitotic figures/square millimetre of tumour tissue in the section (Vesalainen et al, 1995). The results of flow cytometry have been reported in detail previously (Vesalainen et al, 1994). DNA index was available in 182/215 (85%) of cases and S phase fraction in 175/215 (81%) of cases.

### α-Catenin immunohistochemistry

Five-micrometre-thick sections from the primary tumours were deparaffinized, rehydrated and washed twice for 5 min with phosphate-buffered saline (PBS). For α-catenin analysis, the sections were heated in a microwave oven in 0.005 M HCl (pH = 9.7) for 2 × 5 min.

Endogenous peroxidase activity was blocked with 5% hydrogen peroxide for 5 min, followed with a wash for 2 × 5 min with PBS. The tissue sections were incubated overnight at 4°C with a primary mouse monoclonal anti α-catenin antibody (Transduction Laboratories, Lexington, KY, USA) at a working dilution of 1:100. Sections were washed twice for 5 min with PBS and incubated for 30 min using a biotinylated secondary antigen (Vectastain ABC Elite kit, Vector Laboratories, CA, USA) diluted 1:200 in PBS. Slides were washed twice in PBS for 2 × 5 min and incubated for 40 min in preformed avidin–biotinylated peroxidase complexes (Vectastain ABC Elite kit, Vector Laboratories, CA, USA). Sections were washed twice for 5 min with PBS, developed for 5 min with 0.05% 3,3-diaminobenzidine tetrahydrochloride (DAB) (Sigma, UK), slightly counterstained with haematoxylin, dehydrated, cleared and mounted with DePex (BDH, Limited Poole, UK). In each staining batch, normal epithelium served as a positive control. In negative controls, primary antibody was omitted.

### Scoring of immunohistochemistry

The expression of α-catenin in cancer cells was compared with that of normal epithelial cells in the sample. First, the intensity of the staining was scored as follows: negative (0), weak (1) or strong (2). Cancer cells which stained as strong as the normal prostate epithelium were defined as normal expressors (strong intensity). Weak expression was defined as a faint staining clearly weaker than that in normal epithelium. Secondly, the localization of staining signal was categorized into two groups: staining along the cell membranes and cytoplasmic involvement in addition to membranous staining. Finally, the staining signal was graded according to the proportion of positive cancer cells. The fraction of positive cancer cells (%) was primarily analysed in a continuous scale, but for statistical calculations, tumours having over 95% cancer cells positively stained were considered as positive (normal). The others were considered as abnormal. In the scoring process, only well-preserved tumour tissue was evaluated. It became evident that in TURP specimen the chip margins frequently showed no α-catenin signal, probably due to tissue denaturation and coagulation during TURP procedure. To validate the above described scoring principle, a test set of 48 prostatectomy sections were analysed to test the frequency of abnormal α-catenin expression in surgically removed tissues.

### Statistical analysis

In the basic statistical calculations, the SPSS-X program package was used in an IBM computer and the statistical tests used are indicated in connection with the results when appropriate. Univariate survival analysis (log-rank analysis) was based on a life-table method with statistics by Gehan (SPSS-X). Multivariate survival analysis (Cox's analysis) used deaths from prostate cancer as events. Multivariate analysis was done in two phases. The first analysis included TM-categories, Gleason score and the α-catenin indices. The second analysis included all the available parameters.

### RESULTS

Normal and hyperplastic prostate epithelium in the close vicinity of tumours showed strong positive staining along cell membranes throughout the cell–cell boundaries. The intensity of staining was strong (normal) in 190 (88%) tumours (Figure 1A) and weak in 19 (9%) tumours. Six tumours (3%) were completely negative for α-catenin (Figure 1B). Staining pattern along cell membranes was observed in 189 (88%) cases and additional cytoplasmic involvement (Figure 1C) was present in 20 cases (9%). The staining was regarded abnormal (positive tumour cells less than 95% of the total tumour cell population) in 39 (18%) cases. The test set treated by surgical prostatectomy revealed one α-catenin negative case (2%) and seven cases (15%) with less than 95% of positive cells. These figures correspond to those found in the main series of prostate cancer cases.

Both cytoplasmic involvement ($\chi^2 = 89$, $P < 0.001$) and weak staining intensity ($\chi^2 = 59$, $P < 0.001$) were related to abnormal α-catenin expression (positive cells less than 95%). In addition, weak intensity was significantly associated with high grade ($P = 0.006$) and T3–T4 categories ($P = 0.0007$), but not with other analysed cellular features. The negative staining, as well as the cytoplasmic localization of α-catenin, were linked with high T classification, high grade, presence of perineural infiltration, DNA

© Cancer Research Campaign 1999

**Table 1** The significant relationship between the localization of α-catenin signal and various prognostic factors in prostate cancer

| | α-catenin localization in tumour epithelium | | | |
|---|---|---|---|---|
| | Negative | Cytoplasmic and membranous staining | Membranous staining | *P*-value |
| T1 | 0 | 9 | 81 | < 0.001 |
| T2 | 1 | 5 | 40 | |
| T3 | 1 | 12 | 45 | |
| T4 | 4 | 13 | 10 | |
| M0 | 3 | 23 | 131 | 0.009 |
| M1–2 | 3 | 16 | 45 | |
| Gleason score 2–4 | 0 | 2 | 10 | < 0.001 |
| 5–7 | 1 | 6 | 95 | |
| 8–10 | 5 | 31 | 71 | |
| PNI negative | 0 | 10 | 70 | 0.003 |
| positive | 2 | 14 | 36 | |
| M/V index ≤ 7 | 2 | 18 | 114 | 0.002 |
| > 7 | 4 | 21 | 57 | |
| Diploid | 2 | 14 | 91 | 0.008 |
| Aneuploid | 4 | 21 | 56 | |
| S phase ≤ 5% | 1 | 8 | 69 | 0.003 |
| Fraction > 5% | 5 | 27 | 71 | |

PNI = perineural growth; M/V index = volume corrected mitotic index.

**Table 2** The significant relationship between the expression of α-catenin and various prognostic factors in prostate cancer

| | Normal α-catenin expression | Abnormal α-catenin expression | *P*-value |
|---|---|---|---|
| T1 | 81 | 9 | <0.001 |
| T2 | 40 | 5 | |
| T3 | 45 | 12 | |
| T4 | 10 | 13 | |
| M0 | 131 | 23 | 0.05 |
| M1–2 | 45 | 16 | |
| Gleason score 2–4 | 10 | 2 | <0.001 |
| 5–7 | 95 | 6 | |
| 8–10 | 71 | 31 | |
| PNI negative | 70 | 10 | 0.02 |
| positive | 36 | 14 | |
| M/V index ≤ 7 | 114 | 18 | 0.02 |
| > 7 | 57 | 21 | |
| Diploid | 91 | 14 | 0.02 |
| Aneuploid | 56 | 21 | |
| S phase ≤ 5% | 69 | 8 | 0.004 |
| Fraction > 5% | 71 | 27 | |

M/V index = volume corrected mitotic index; PNI = perineural growth.

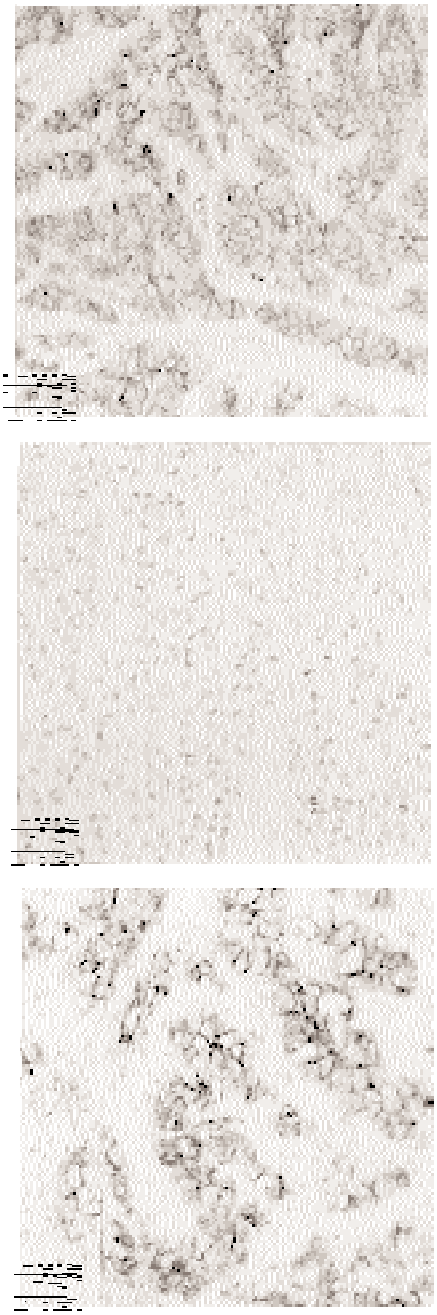

**Figure 1** (**A**) A prostate cancer showing strong normal membranous expression of α-catenin (magnification 250x). (**B**) A poorly differentiated α-catenin-negative prostate cancer (magnification 250×). (**C**) A poorly differentiated prostate cancer shows both cytoplasmic and membranous staining pattern of α-catenin (magnification 250×)

aneuploidy, high mitotic index, high S phase fraction and metastasis (Table 1). The reduced expression of α-catenin (positive cells less than 95%) was related to several prognostic factors as detailed in Table 2. In the test set of prostate cancers, all the cases with abnormal α-catenin expression belonged to pT3A–pT3C categories, except one case which was pT4. The Gleason score was over 6 in six of the cases with abnormal α-catenin expression.

In the univariate survival analysis, cytoplasmic signal in addition to membranous staining predicted unfavourable patient outcome (Figure 2). The reduced expression was associated with lowered survival probability in the entire series with a borderline significance (Figure 3). However, it had no statistically significant prognostic value either in M0 ($P$ = 0.2) or in T1–2 M0 tumours (Figure 4).

In the multivariate analysis including TM-classification, Gleason score and α-catenin indices, abnormal α-catenin staining had independent prognostic value only in T1–2 M0 tumors (Table 3). Recurrence-free survival of M0 tumors was independently related to T-category (relative risk (RR) = 1.74, 95%

© Cancer Research Campaign 1999 

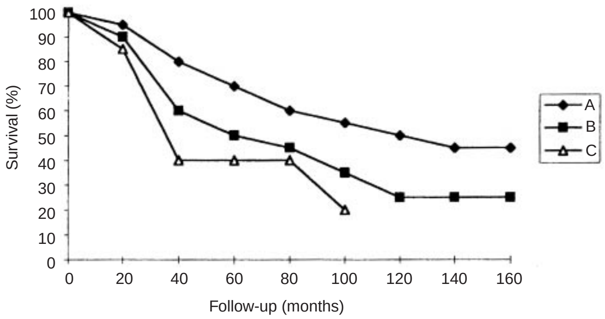

**Figure 2** The survival of patients categorized according to the localization of α-catenin signal ($P = 0.04$, $\chi^2 = 6$). Curve A: membranous staining, $n = 188$; Curve B: membranous and cytoplasmic staining pattern, $n = 20$; Curve C: negative staining, $n = 6$

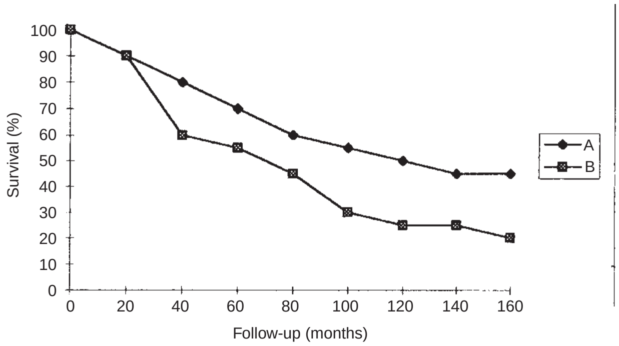

**Figure 3** The survival of patients categorized according to the expression of α-catenin. The curves are almost significantly separated ($P = 0.06$, $\chi^2 = 3.5$). Curve A: α-catenin normal, $n = 176$; Curve B: α-catenin abnormal, $n = 39$

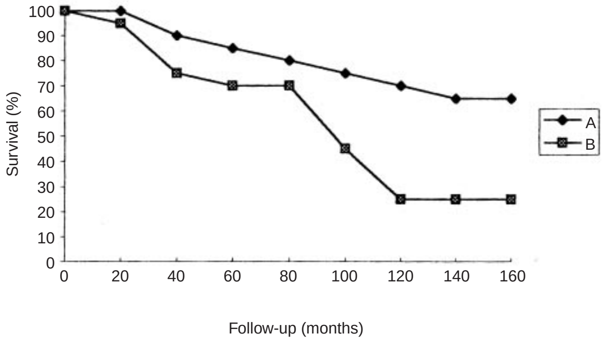

**Figure 4** The survival of patients with a T1–2 M0 tumour categorized according to the expression of α-catenin ($P = 0.1$, $\chi^2 = 2.6$). Curve A: α-catenin normal, $n = 121$; Curve B: α-catenin abnormal, $n = 14$

confidence interval (CI) 1.27–2.39, $P < 0.001$), DNA aneuploidy (RR = 3.41, 95% CI 1.74–6.69, $P < 0.001$) and the staining intensity of α-catenin (RR = 0.51, 95% CI 0.29–0.89, $P = 0.017$). If all the analysed features (T-category, presence of metastases, Gleason grade, perineural infiltration, mitotic index, DNA ploidy and S phase fraction) were entered in the model, independent prognostic factors in the entire series were M-category (RR = 3.12, 95% CI 1.78–5.46, $P = 0.0002$), T-category (RR = 1.75, 95% CI

**Table 3** The independent prognostic factors in the entire cohort and in non-metastatic as well as in clinically local tumours

| | β (s.e.) | *P*-value | RR (95%CI) |
|---|---|---|---|
| T1–4 M0–1 tumours | | | |
| M-category | 1.11 (0.25) | < 0.001 | 3.04 (1.83–5.04) |
| T-category | 0.46 (0.13) | < 0.001 | 1.59 (1.22–2.07) |
| Gleason score | 0.55 (0.25) | 0.026 | 1.74 (1.06–2.85) |
| T1–4 M0 tumours | | | |
| T-category | 0.59 (0.16) | < 0.001 | 1.81 (1.32–2.49) |
| Gleason score | 0.75 (0.34) | 0.028 | 2.12 (1.08–4.14) |
| T1–2 M0 tumours | | | |
| T1 vs T2 | 1.73 (0.40) | < 0.001 | 5.68 (2.57–12.56) |
| α-Catenin expression | –1.06 (0.51) | 0.037 | 0.34 (0.12–0.93) |

1.33–2.29, $P = 0.0001$) and M/V index (RR = 1.02, 95% CI 1.00–1.05, $P = 0.041$). In M0 tumours, independent predictors were T-category (RR = 1.59, 95% CI 1.12–2.24, $P = 0.005$) and Gleason score (RR = 3.30, 95% CI 1.43–7.60, $P = 0.005$). Finally, in clinically local T1–2 M0 tumours, Gleason score (RR = 3.08, 95% CI 1.15–8.21, $P = 0.024$) and M/V index (RR = 1.06, 95% CI 1.01–1.12, $P = 0.009$) were independent prognostic factors of survival.

### DISCUSSION

Normal and hyperplastic prostatic epithelium revealed normal, strong and uniform staining pattern of α-catenin, whereas in cancer tissue various α-catenin staining signals were frequently detected. The α-catenin expression was abnormal in 18% of the cases in the main series. Consequently, 15% of cases in the test set treated by prostatectomy showed similar reduced α-catenin signal. In a heterogenous group of prostate cancers, Richmond and co-workers (Richmond et al, 1997) reported up to 41% of the tumours (33/79 cases) showing abnormal α-catenin expression which is a higher figure than ours. Another previous study with local incidentally detected tumours reported that the proportion of abnormal α-catenin expression was over 40% (10/23 cases) (Crundwell et al, 1997). Both of these studies have utilized TURP specimens for immunohistochemistry but no description is given whether all the tumour tissue was used in the scoring process. According to our experience, it is highly important to exclude the TURP chip margins from evaluation since the tissue damage due to heating seems to abolish α-catenin immunoreactivity. Additional support for this comes from our test set (surgical prostatectomy specimens) which showed a similar frequency distribution of α-catenin as the main series. We believe that this notion seems to validate the adopted scoring principle. Similar results have also been reported by Umbas and co-workers (Umbas et al, 1997), since they observed aberrant α-catenin expression in 52% of the heterogeneous group of prostate tumours, while in their small radically operated group of tumours only 20% (4/20) of the cases showed abnormal α-catenin expression (Umbas et al, 1997).

There was a significant relationship between the reduced α-catenin expression and high Gleason score, suggesting more aggressive behaviour and increased potential to spread locally and to distant sites. A similar relationship between high grade and α-catenin abnormalities has been previously reported both in prostate cancer (Richmond et al, 1997) and in other epithelial

© Cancer Research Campaign 1999

neoplasms (Rimm et al, 1995; Shimazui et al, 1997; van der Wurff et al, 1997). α-catenin abnormalities were frequently detected in tumours showing perineural growth which indicates that α-catenin abnormalities also favour invasive growth in vivo. The significant relationship between α-catenin abnormalities and all proliferation indices found in this study is in line with the inter-relationship between Gleason score and α-catenin abnormalities (Richmond et al, 1997).

The presence of metastasis and local spread were related to reduced α-catenin expression. Abnormalities in the staining found in our T1–T2 tumours were rare but the frequency clearly increased in T3–T4 tumours. Similarly, in the test set of tumours treated by prostatectomy, the final histopathological analysis of the prostatectomy specimens (pT) revealed that all the tumours showing down-regulation of α-catenin belonged mostly to pT3A–C categories. These results suggest that tumours with reduced α-catenin expression rapidly progress locally into invasive disease. It is obvious that the metastatic potential of prostate tumours is not dependent on cell adhesion factors alone, and many other factors including the host response are involved (Vesalainen et al, 1994). In alignment with our notion, the relationship between α-catenin expression and tumour spread has been reported previously in prostate cancer (Richmond et al, 1997) and in tumours of the urinary tract (Shimazui et al, 1996).

The earlier studies in prostate cancer indicate that α-catenin abnormalities predict unfavourable prognosis (Richmond et al, 1997; Umbas et al, 1997). In our study, abnormal signal localization as well as down-regulation of α-catenin were related to unfavourable prognosis supporting these previous findings (Richmond et al, 1997; Umbas et al, 1997).

Since several genetic changes occur simultaneously in prostate cancer (Isaacs, 1995), only multivariate analysis of prognostic factors can give insight to the impact of individual prognostic factors on patient survival. In the current study abnormal α-catenin expression was an independent prognosticator only in T1–2 M0 group of tumours and when Gleason score, T-classification and different α-catenin indices were included in the analysis. In a subgroup analysis of M0 cases, reduced α-catenin signal was independently related to early recurrence. Umbas and co-workers (Umbas et al, 1997) reported that all four cases out of 20 patients treated by radical prostatectomy and having aberrant expression of α-catenin recurred, while those tumours with normal expression had no recurrence within 40 months follow-up. In another study α-catenin had significant prognostic value in terms of recurrence-free survival but α-catenin expression was not related to survival (Crundwell et al, 1997). In the study by Richmond and co-workers (Richmond et al, 1997) α-catenin was an important prognostic factor only in the univariate analysis. Umbas et al (1997) also observed that α-catenin was a predictor of patient outcome in advanced disease and recurrence-free survival in radically treated local tumours. However, they have not described the accuracy of preoperative TNM in comparison to final post-operative pTNM classification. Although the disease progression was associated with abnormal α-catenin expression, the question still remains whether α-catenin is an independent predictor of progression. The data in localized renal cell carcinoma suggest that α-catenin, indeed, seems to have independent prognostic value (Shimazui et al, 1997).

In conclusion, abnormal α-catenin expression is related to advanced disease stages and malignant cellular features in prostate cancer and the analysis of α-catenin expression may give additional prognostic information in M0 prostate cancer.

### ACKNOWLEDGEMENTS

This study was financially supported by a research grant (EVO funding) from the Kuopio University Hospital. Urologist Satu Vesalainen is gratefully acknowledged for her contribution in the initial phases of this study. The technical assistance of MRs Aija Parkkinen is gratefully acknowledged.

### REFERENCES

Behrens J, Mareel MM, Van Roy FM and Birchmeier W (1989) Dissecting tumor cell invasion: epithelial cells acquire invasive properties after loss of uvomorulin-mediated cell–cell adhesion. *J Cell Biol* **108**: 2435–2447.

Breen E, Steele GJ and Mercurio AM (1995) Role of the E-cadherin/alpha-catenin complex in modulating cell–cell adhesive properties of invasive colon carcinoma cells. *Ann Surg Oncol* **2**: 375–385.

Crundwell MC, Arkell DG, Gearty J and Phillips SMA (1997) Genetic alterations in incidentally diagnosed, transitional zone prostate cancer: a seven-year follow-up. *J Urol* **158**: 1568–1575.

Frixen UH, Behrens J, Sachs M, Eberle G, Voss B, Warda A, Löchner D and Birchmeier W (1991) E-cadherin mediated cell–cell adhesion prevents invasiveness of human carcinoma cells. *J Cell Biol* **113**: 173–185

Gleason DF (1997) Histological grading and clinical staging of prostate carcinoma. In *Urologic Pathology*: *The Prostate*, p. 171. Philadelphia: Lea & Febiger

Gumbiner BM and McCrea PD (1993) Catenins as mediators of the cytoplasmic functions of cadherins. *J Cell Sci* **17**: 155–158.

UICC (International Union Against Cancer) (1978) *TNM Classification of Malignant Tumors*. Geneva: UICC

Isaacs WB (1995) Molecular genetics of prostate cancer. *Cancer Surv* **25**: 357–379

Morton RA, Ewing CM, Watkins JJ and Isaacs WB (1995) The E-cadherin cell–cell adhesion pathway in urologic malignancies. *World J Urol* **13**: 364–368

Morton RA, Ewing CM, Nagafuchi A, Tsukita S and Isaacs WB (1993) Reduction of E-cadherin levels and deletion of alpha-catenin gene in human prostate cancer cells. *Cancer Res* **53**: 3585–3590

Murant SJ, Handley J, Stower M, Reid N, Cussenot O and Maitland NJ (1997) Co-ordinated changes in expression of cell adhesion molecules in prostate cancer. *Eur J Cancer* **33**: 263–271

Paul R, Ewing CM, Jarrard DF and Isaacs WB (1997) The cadherin cell–cell adhesion pathway in prostate cancer progression. *Br J Urol* **79**: 37–43

Richmond PJM, Karayiannakis JA, Nagafuchi A, Kaisary AV and Pignatelli M (1997) Aberrant E-cadherin and α-catenin expression in prostate cancer: correlation with patient survival. *Cancer Res* **57**: 3189–3193

Rimm DL, Sinard JH and Morrow JS (1995) Reduced α-catenin and E-cadherin expression in breast cancer. *Lab Invest* **72**: 506–512

Shimazui T, Brinquier PP, van Berkel H, Ruijter E, Akaza H, Debruyne FMJ, Oosterwijk E and Schalken JA (1997) Decreased expression of alfa-catenin is associated with poor prognosis of patients with localized renal cell carcinoma. *Int J Cancer* **74**: 523–528

Shimazui T, Schalken JA, Giroldi LA, Jansen CF, Akaza H, Koisto K, Debruyne FM and Brinquier PP (1996) Prognostic value of cadherin-associated molecules (α-, β- and γ-catenins and p120cas) in bladder tumors. *Cancer Res* **56**: 4154–4158

Umbas R, Isaacs WB, Brinquier PP, Schaafsma HE, Karthaus HFM, Oosterhof GON, Debruyne FMJ and Schalken JA (1994) Decreased E-cadherin expression is associated with poor prognosis in patients with prostate cancer. *Cancer Res* **54**: 3929–3933

Umbas R, Isaacs WB, Brinquier PP, Xue Y, Debruyne FMJ and Schalken JA (1997) Relation between aberrant α-catenin expression and loss of E-cadherin function in prostate cancer. *Int J Cancer* **74**: 374–377

Van der Wurff A, Vermeulen SJ, van der Linden EP, Mareel MM, Bosman FT and Arends JW (1997) Patterns of α- and β-catenin and E-cadherin expression in colorectal adenomas and carcinomas. *J Pathol* **182**: 325–330

Vermeulen S, van Marck V, van Hoorle L, van Roy F, Bracke M and Mareel M (1996) Regulation of the invasion suppressor function of the cadherin/catenin complex. *Pathol Res Pract* **192**: 694–707

Vesalainen S, Lipponen P, Talja M and Syrjänen K (1995) Mitotic activity and prognosis in prostatic adenocarcinoma. *Prostate* **26**: 80–86

© Cancer Research Campaign 1999 

Vesalainen S, Nordling S, Lipponen P, Talja M and Syrjänen K (1994) Progression and survival in prostatic adenocarcinoma: a comparison of clinical stage, Gleason score, S-phase fraction and DNA ploidy. *Br J Cancer* **70**: 309–314
Vleminckx K, Vakaet L, Mareel M, Fiers W and van Roy F (1991) Genetic manipulation of E-cadherin expression by epithelial tumor cells reveals an invasion suppressor role. *Cell* **66**: 107–119
Zsciesche W, Schonborn I, Behrens J, Herrenknecht K, Hartveit F, Lilleng P and Birchmeier W (1977) Expression of E-cadherin and catenins in invasive mammary carcinomas. *Anticancer Res* **17**: 561–567

*© Cancer Research Campaign 1999*
